# Seeded Ising Model and Distributed Biometric Template Storage and Matching

**DOI:** 10.3390/e23070849

**Published:** 2021-07-01

**Authors:** Hyeong In Choi, Sungjin Lee, Hwan Pyo Moon, Nam-Sook Wee, Daehoon Kim, Song-Hwa Kwon

**Affiliations:** 1 Department of Mathematics, Seoul National University, Seoul 08826, Korea; 2 iSciLab Corporation, Seoul 08791, Korea; hichoi@iscilab.com; 3 Department of Mathematics, Daejin University, Pocheon 11159, Korea; hyper@daejin.ac.kr; 4 Department of Mathematics, Dongguk University-Seoul, Seoul 04620, Korea; hpmoon@dongguk.edu; 5 Division of Smart Management, Hansung University, Seoul 02876, Korea; nswee@hansung.ac.kr; 6 IriTech, Inc., Fairfax, VA 22030, USA; dhkim@iritech.com; 7 Department of Mathematics, The Catholic University of Korea, Bucheon 14662, Korea

**Keywords:** Ising model, biometric template, partial template, distributed biometrics

## Abstract

It is known that a variant of Ising model, called *Seeded Ising Model*, can be used to recover the information content of a biometric template from a fraction of information therein. The method consists in reconstructing the whole template, which is called the *intruder template* in this paper, using only a small portion of the given template, a *partial template*. This reconstruction method may pose a security threat to the integrity of a biometric identity management system. In this paper, based on the Seeded Ising Model, we present a systematic analysis of the possible security breach and its probability of accepting the intruder templates as genuine. Detailed statistical experiments on the intruder match rate are also conducted under various scenarios. In particular, we study (1) how best a template is divided into several small pieces called partial templates, each of which is to be stored in a separate silo; (2) how to do the matching by comparing partial templates in the locked-up silos, and letting only the results of these intra-silo comparisons be sent to the central tallying server for final scoring without requiring the whole templates in one location at any time.

## 1. Introduction

**Figure 1:**
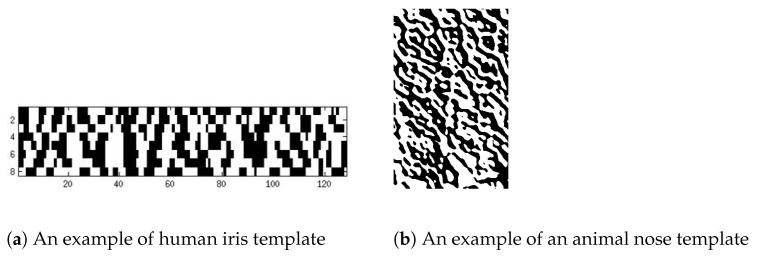
Examples of templates.

Most, if not all, biometric algorithms have a common thread of steps. The first step is the generation of a secondary data structure that is usually called the template. Then, the identity recognition is done by comparing the templates in question. For example, in the case of human iris recognition, the Gabor sine and cosine transforms are used to generate the templates [1,2,3,4,5,6]; similarly, for animal nose pattern recognition, a similar Gabor transform is used [7]. Figure 1a shows an example of a human iris template, and Figure 1b shows that of an animal nose image. Each of these templates is a two-dimensional array of 0 s (black) and 1 s (white). One salient characteristic one can easily discern from these templates is that the 0 s and 1 s are clustered together to form a certain coherent structure that is supposed to be responsible for each individual’s biometric unique pattern.

C.Y. Han and the authors of this paper—Choi, Lee, Wee and Kwon—have studied this clustering phenomenon in light of the Ising model [8]. In it, they proposed a new Ising model, called the Seeded Ising Model, which is a variant of the Ising model. In this model, certain bits are fixed while other bits are allowed to change according to the Ising Model dynamics. Biometric patterns such as iris and nose pattern are the result of the embryonic development of mesoderm and ectoderm with the initial condition, which is presumed to be random. The Seeded Ising Model proposed in [8] can be viewed as a mathematical abstraction of the biometric pattern formation in which the randomly chosen seeds represent the random initial condition and the pattern formation is modeled as an Ising model’s dynamic process. They have discovered several interesting results, one of which is what they call the *effective statistical degree of freedom*. In particular, they showed, using the Seeded Ising Model dynamic evolution, that if the total number of bits in the template is 2048, one only needs 342 bits as seeds to recover quite faithfully the information content of the original template. They showed this result by devising what they call the reconstructed template, which is an artificial template constructed using only a small number of seeds. This reconstructed template opens a door to a would-be attacker in that only a small portion of template information is needed for the identity fake. The purpose of this paper is to study in detail how such security hole may occur and how one can prevent such a security breach from the point of view of the Seeded Ising Model.

There are several studies addressing the security issues related to the biometric templates. Among the notable are the approaches of cancelable biometrics and the schemes of biometric cryptosystem. In the methods of cancelable biometrics, biometric templates are transformed by one-way function, and the transformed templates are stored and served as a means of identifiers [9,10,11,12,13,14,15,16]. (For an extensive survey, see [17].) With cancelable iris templates [14,15,16], biometric information would be kept securely even when the cancelable iris templates are leaked, since biometric information can not be recovered from cancelable iris templates. In the methods of biometric cryptosystems, transformed biometric templates are combined with cryptographic keys to generate secure templates [18,19,20].

On the other hand, various biometric algorithms using watermarking [21,22,23,24,25,26] have also been proposed to secure biometric data. In the scheme proposed by Abdullah et al. [26], iris templates are divided into two shares, and one share is stored in the database, whereas the other share is stored on user’s smart card. When it comes to comparing iris templates, two shares are combined into the original iris template beforehand.

As an alternative approach for enhancing the safety and security of biometric data, some national governments and financial institutions are now beginning to devise a new scheme of dividing the biometric templates and storing the divided partial templates in separate locations. The recent initiative of the Korea Financial Telecommunication & Clearing Institute, a governmental agency of Korea, is one such example. However, many fundamental issues concerning this scheme are not fully studied. For example, consider the following: into how many partial templates should the whole template be divided to ensure the integrity if some storage location were to be hacked and the stored partial templates are leaked? Moreover, there are other theoretical and practical issues related to this kind of division scheme.

In this paper, we systematically study the following problems: how to divide a biometric template into several partial templates and how many such partial templates are needed to guarantee the integrity of the system; how to store such partial templates in separate silos and how to match such partial templates in the confine of each silo; and how to combine the matching results of partial templates without requiring the reconstruction of the whole template in a central location. In this paper, we use iris templates as an illustrative example of our methods, but the same can be applied to other biometrics such the animal nose biometrics. For the basics of iris recognition algorithms, refer to [1,2,3,4,5,6]. In particular, as is the standard practice in iris recognition, the iris template is generated by applying the Gabor sine and cosine transforms.

The matching between two templates *A* and *B* is done by computing the following Hamming distance, which is also called the dissimilarity score:

$$d_{H}\left(A,B\right)=\frac{\left|\right|\left(C_{A}⊗C_{B}\right)\cap M_{A}\cap M_{B}\left|\right|}{\left|\right|M_{A}\cap M_{B}\left|\right|},$$

where $C_{A}$ and $C_{B}$ are the phase codes of *A* and B, respectively; $M_{A}$ and $M_{B}$ are the occlusion masks of *A* and B, respectively; and ⊗ is the bitwise Boolean Exclusive-OR operator, whereas ∩ is the bitwise AND operator and ||·|| is the norm of a bit vector. For more details, refer to [1,2,3,4,5,6].

## 2. Distributed Template Storage and Matching scheme

The iris template is a two-dimensional array of complex binary numbers. By a complex binary number, we mean a complex number whose real and imaginary parts are 0 s and 1 s. By taking the real and imaginary parts separately, the iris template can be thought of as a pair of two-dimensional array of 0 s and 1 s of the same dimension. These real and imaginary parts come from the Gabor transform. However, for the simplicity of discussion, we take for the template the real part of the two-dimensional array. (There is no preference for the real part, and one may just as well take the imaginary part. It should be pointed out that we assume that real and imaginary parts are used independently in full implementation. In this sense, it is not congruent with the dominant iris2pi like algorithms. However, with slight modification of the algorithm, our proposed method still remains valid.) An auxiliary information that goes with the template is the occlusion mask, which is also a two-dimensional array of 0 s and 1 s of the same dimension. The occlusion mask contains the information on which parts of the iris is occluded by something other than the iris such as eyelids, eyelashes, specular reflection, and so on that act as a hindrance to the correct matching.

**Figure 2:**
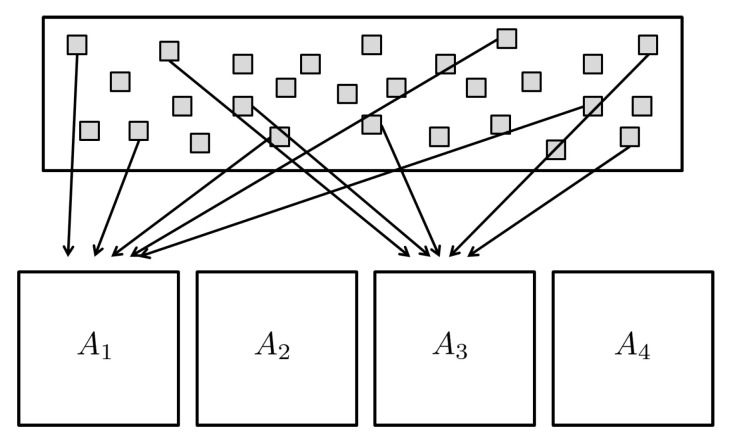
Dispersed-form division.

The basic scheme we use in this paper is to divide the template into several smaller units, called *partial templates*, and to store and compare them separately. The ways in which the template is divided can be diverse. One way of division is to divide a template into predetermined rectangular blocks, which we call the *block-form* division. Figure 2, on the other hand, shows a different manner in which a template is divided into partial templates. Unlike the block-form division, each partial template in this case is a collection of dispersed entries of the original template. For this reason, we call this way of dividing the template into partial templates a *dispersed-form* division. In this case, though, there are two different ways of creating partial templates: one way is to choose for each partial template the entries of the original template that contains only 0 s or 1 s; the other is to choose the entries without regard to its value of 0 s and 1 s. The former is called the *Z-dispersion* and the latter *R-dispersion*. It should be noted that the occlusion mask is also divided in exactly the same manner as the template is divided into partial templates. Since each partial template is stored separately, we need to keep track of which entry of the original template each element of a partial template corresponds to. In fact, from now on, by a *partial template*, we mean the collection of these three ingredients: *divided template, occlusion mask divided accordingly and the information with regard to the entry position on the original template*.

### 2.1. Storage of Partial Templates

Our partial template storage scheme is that after a whole template is divided, each of the resulting partial templates is stored in a different location. The different location could mean physically distant location, or it can be a logically separate location such that even if one location is attacked by a hacker, the other locations still remain relatively safe. The question then is whether the whole identity verification system is still integrally safe even if one partial template is leaked out.

### 2.2. Comparison

The conventional method of comparison requires comparing two whole templates (e.g., a biometric probe and a biometric reference). However, if the hacker gets hold of the biometric server performing the comparison, he/she can steal such templates. Normally the biometric server performing the comparison and template database are well guarded to prevent such mishaps. Nonetheless, such attacks do happen, and if so, the consequence is dire because biometric data, unlike passwords, cannot be revoked. Such concern has been one of the key reasons why there has been strong resistance against the wide-spread acceptance of biometric national ID. However, with the proliferation of identity theft and fraud, the world is gradually moving toward biometric IDs. The question is how to provide safeguard it in such an environment. Our scheme is designed to address such problem.

There are many national or semi-national entities that are keenly aware of the danger of whole-sale template hacking. For instance, for this reason, Korea Financial Telecommunications & Clearings Institute requires the participating biometric vendors to divide the biometric references and store the divided biometric references in separate locations. Although the divided biometric references are stored in separate locations, when it comes to comparison, they perform the comparison on the whole biometric reference: the central biometric server has to collect the divided biometric references from all of the storage locations and stitch them together to create a whole biometric reference and then do the comparison using the whole biometric reference.

**Figure 3:**
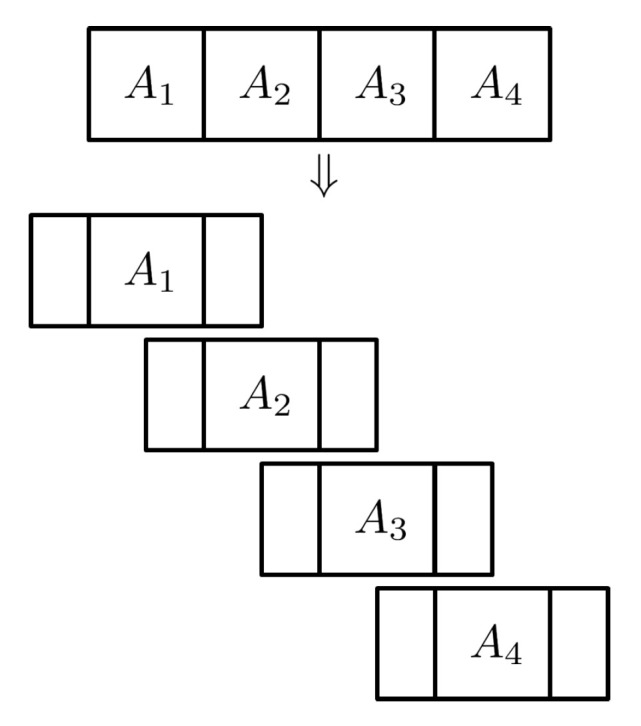
Padded partial templates.

**Figure 4:**
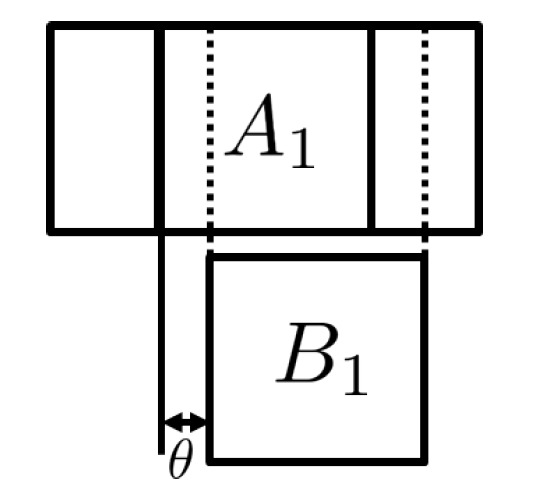
Partial template comparison with rotation angle θ.

In this paper, we propose a novel scheme that avoids such stitching in a central location. It works as follows. When a biometric probe arrives at the biometric server, it is divided into partial templates with some padding, called a *padded partial template*, to accommodate for the rotation angle variation. (Figure 3 briefly illustrates how to generate padded partial templates from a biometric template.) Then, such a padded partial template is sent to the appropriate storage location. The storage location has its own local biometric server, and this local biometric server compares the sent-down padded partial biometric probe with the partial biometric reference. This comparison process is called a *partial comparison*. Figure 4 shows how partial comparison is done for a rotation angle θ. For each angle, the resulting partial dissimilarity score is computed and forwarded to the central biometric server. The central biometric server then collects all the partial dissimilarity scores per angle and computes the dissimilarity score of the whole template per angle. The final dissimilarity score is the minimum of the dissimilarity scores taken for all angles. It should be noted that the central biometric server is not actually doing any comparison per se. Rather, it is serving as an arbiter based on the partial dissimilarity scores per angle. It should also be noted that the partial templates once stored in a storage location will never leave that location, and all the computation is done inside that storage location.

When the fractional Hamming distance $d_{H}$ is used as a dissimilarity score, the explicit formula for partial comparison is as follows. The dissimilarity score d(A,B) between a biometric probe *A* and a biometric reference *B* is given by

$$\begin{array}{c} d\left(A,B\right)=\min_{1\leq j\leq k}d_{\theta_{j}}\left(A,B\right), \end{array}$$

where $\theta_{1},\cdots ,\theta_{k}$ are rotation angles to be allowed in comparison. Suppose that $B_{1},\cdots ,B_{m}$ denote all the partial biometric templates from a biometric reference B, where *m* is the number of local biometric servers. Then, $d_{\theta_{j}},$ the dissimilarity score between *A* and *B* with rotation angle $\theta_{j},$ is computed by

$$d_{\theta_{j}}\left(A,B\right)=\frac{\sum_{i=1}^{m}a_{ij}d_{ij}}{\sum_{i=1}^{m}a_{ij}},$$

where $d_{ij}$ is the partial dissimilarity score between the padded partial template from *A* and the partial biometric reference $B_{i}$ with angle $\theta_{j}$ computed at the *i*-th local biometric server, $a_{ij}$ is the number of bits whose masking bit value is 1 in both of the padded partial template and the partial biometric reference with angle $\theta_{j}.$

It should also be noted that the padding and unpadding decision can be reversed. Specifically, one may store padded partial templates in the storage location and create unpadded partial templates from the biometric probe and send down such unpadded template to each storage location. The rest of the comparison proceeds the same way. Which one is more preferable depends on the trade-off between storage efficiency and the safety of the biometric probe.

## 3. Occlusion Attack

Suppose a partial biometric reference is leaked out from a system with distributed storages of divided partial biometric references as this paper is proposing. Then, the most simple attack with the leaked partial biometric reference would be the so-called “occlusion attack” by which one may submit to the iris recognition system the leaked partial biometric reference with the intentionally modified occlusion mask of which every bit value is marked as a damaged or occluded bit; i.e., the value of 0 except the bits contained in the leaked partial biometric reference. If the partial template *p* of a full template *x* is leaked out and the biometric reference *x* still remains in the system, then this occlusion attack would be always successful since the Hamming distance between *x* and *p* with the modified mask would be zero. Even if the template *x* is replaced by another template $x^{\prime }$ of the same person, this occlusion attack would still be successful with high probability, since the Hamming distance between $x^{\prime }$ and *p* with the modified occlusion mask is very close to the Hamming distance between *x* and $x^{\prime },$ which would be less than the given threshold of the system with very high probability. Thus, the distributed storage of templates alone does not provide the necessary safety measure when any partial biometric reference is leaked out. To protect the system against this occlusion attack, the system must utilize a new distance measure that penalizes the occlusion bits. For this purpose, we introduce a novel distance, the modified fractional Hamming distance, or simply the modified Hamming distance $d_{H}^{\prime }$ given by

$$\begin{array}{ccc} d_{H}^{\prime }\left(A,B\right) & = & \frac{\left|\right|\left(C_{A}⊗C_{B}\right)\cap M_{A}\cap M_{B}\left|\right|}{N}\\ & & +\frac{0.5||∼(M_{A}\cap M_{B})||}{N} \end{array}$$

with the consideration of how many valid (i.e., mask bit value 1) bits two templates *A* and *B* have in common. In the definition of the modified fractional Hamming distance $d_{H}^{\prime },$∼ is the bitwise NOT operator, and *N* denotes the number of bits in a phase code. The basic idea of this new distance is to give the dissimilarity score of 0.5 for each invalid (i.e., mask bit value 0) bit.

The modified Hamming distance $d_{H}^{\prime }$ is related with the Hamming distance $d_{H}$ through the equation:

(1) $$\begin{array}{ccc} d_{H}^{\prime }\left(A,B\right) & = & 0.5-\left(0.5-d_{H}\left(A,B\right)\right)\frac{l}{N}, \end{array}$$

where $l=\left|\right|M_{A}\cap M_{B}\left|\right|$ is the number of bits that are actually compared between two templates *A* and B.

**Figure 5:**
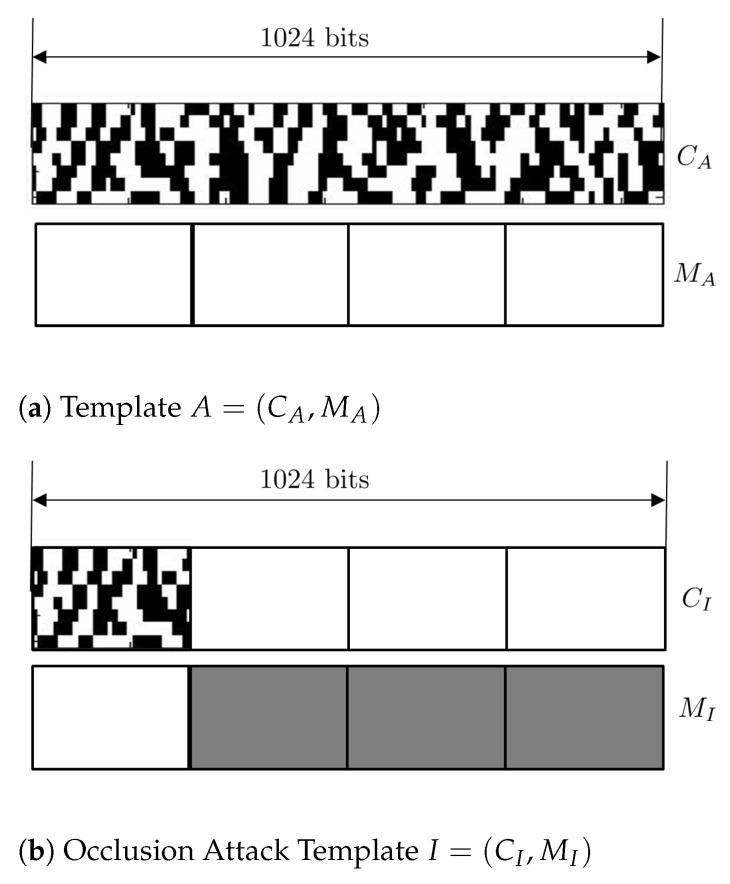
A template with no occlusion and an intruder template for an occlusion attack.

For an illustration of how differently $d_{H}$ and $d_{H}^{\prime }$ behave, we suppose that $d_{H}\left(A,A^{\prime }\right)=0.2$, where templates *A* and $A^{\prime }$ of length 1024 have no occlusion at all, and a partial template p, whose length is 256, of $A^{\prime }$ is leaked out. In an occlusion attack, an intruder template $I=(C_{I},M_{I})$ from the leaked partial template *p* is constructed with the mask $M_{I}$, whose bit values are 0 except where phase bit is available. For schematic diagrams of templates *A* and *I*, see Figure 5. Then, it is on the average that $d_{H}\left(A,I\right)=0.2.$ This means that the Hamming distance of the intruder template is the same as that of the original template; hence, the occlusion attack should be successful. On the other hand, $d_{H}^{\prime }\left(A,I\right)=0.2\times 0.25+0.5\times 0.75=0.425.$ This higher number implies that the occlusion attack is more likely to fail (see Table 3). Thus, an occlusion attack would be successful with high probability when the fractional Hamming distance is employed, while with the modified Hamming distance, it is much less likely to succeed.

We shoud note that some commercial implementations of iris comparison enforce a minimum number of unmasked bits in the code in order to make a recognition decision. Such a requirement on the number of available blocks of iris codes can be a practical remedy to the occlusion attack.

## 4. Seeded Ising Model and Intruder Template

Besides occlusion attacks, the next plausible attack would be the reconstruction of iris templates based on a leaked partial biometric reference, so that the reconstructed templates are very close to real human templates of the same person whose partial biometric reference is leaked. For such attacks to succeed, one may need a model of human iris templates, which explains how human iris templates would be generated. Han et al. [8] investigated the statistical natures of human iris templates and proposed the *Seeded Ising Model*. This is a variant of the Ising model [27] where bits in given locations stay fixed throughout the Ising model dynamic evolution. With this Seeded Ising Model, a full template can be reconstructed from partial information of the whole template. In contrast to iris image reconstruction from full biometric templates [28], iris templates, not iris images, can be reconstructed through the Seeded Ising Model from partial template information. In the subsequent paragraph, we briefly restate the Seeded Ising Model proposed in [8] to fix notational conventions for template representation and template reconstruction.

First, a real or imaginary part of the human iris template is modeled by a binary random field *x* on an $q_{1}\times q_{2}$ regular lattice. Although each bit of iris template is binary with value 0 or 1, when it comes to the Seeded Ising Model presentation, we use the convention that each bit has a value −1 or 1, instead. Therefore according to this convention, 0 in an iris template is replaced with −1 for the Ising model and vice versa. With this notational convention, the space of all iris templates is denoted by

$$T=\{x|x_{i,j}\in \left\{-1,1\right\}\;\mathrm{for}\;1\leq i\leq q_{1},\;\;1\leq j\leq q_{2}\}.$$

To simplify the representation of the position in a $q_{1}\times q_{2}$ regular lattice, we use the univariate indexing scheme by utilizing a mapping, for example, $\left(i,j\right)\mapsto k=i+\left(j-1\right)\times q_{1}.$ With the univariate indexing scheme, the space of iris templates can be simply written by $T=\{x|x_{k}\in \left\{-1,1\right\}\;\mathrm{for}\;1\leq k\leq q_{1}q_{2}\}.$

For a given subset $I\subseteq \{1,2,\cdots ,q_{1}q_{2}\},$ a map s:I→{−1,1} is regarded as “partial template data” specifying the value of the template at positions in I. Therefore, the set of templates that have the same partial template data as *s* is denoted by $T\left(s\right)=\{x|x_{k}=s\left(k\right)\;\mathrm{for}\;\mathrm{each}\;k\in I\}.$

For a given seed s, we model the probability distribution P(x) on the space of T(s) by

$$P\left(x\right)=\frac{1}{Z}\exp\left(\sum_{i∼j}J_{i,j}x_{i}x_{j}\right),$$

where i∼j means the positions *i* and *j* are adjacent to each other; thus, the sum is done over all adjacent positions; $J_{i,j}$ is a constant parameter for the adjacent positions *i* and *j*; and *Z* is the partition function given by

$$Z=\sum_{x\in T(s)}\exp\left(\sum_{i∼j}J_{i,j}x_{i}x_{j}\right).$$

Since we use different $J_{i,j}$ depending on whether *i* and *j* are horizontally or vertically adjacent to each other, we say the relation $i{∼}_{v}j$ means that two positions *i* and *j* are vertically adjacent to each other, and the relation $i{∼}_{h}j$ that two positions *i* and *j* are horizontally adjacent to each other with circular-end conditions employed for each row of a template. Note that we think of the first column and the last column in a regular lattice to be adjacent to each other with circular-end conditions employed. In this paper, we set $J_{i,j}=J_{v}$ when two positions *i* and *j* are adjacent vertically, and $J_{i,j}=J_{h}$ when two positions *i* and *j* are adjacent horizontally. With these conventions, P(x) can be written as

$$P\left(x\right)=\frac{1}{Z}\exp\left(J_{v}\sum_{i{∼}_{v}j}x_{i}x_{j}+J_{h}\sum_{i{∼}_{h}j}x_{i}x_{j}\right).$$

This probabilistic model of the space of templates T(s) with seed *s* is called the *Seeded Ising Model.*

In [8], the best parameter of $J=(J_{v},J_{h})$ was found to be $J=\left(J_{v},J_{h}\right)=\left(0.2,0.3\right),$ and we use the same value for the parameter *J* in this paper as well.

We will now discuss how to reconstruct iris templates when only a partial template *s* is available. In this paper, the templates reconstructed from a partial template will be called *Intruder Templates* since we are supposing the case in which the partial template is leaked out, and reconstructed templates are used to intrude into the iris recognition system. By the partial template s, we mean partial information of a template x, which tells us the locations of available bit values of *s* in *x* as well as bit values of s. Thus, we can treat a partial template *s* as a function s:I→{−1,1}, where $I\subseteq \{1,2,\cdots ,q_{1}q_{2}\}$ is the set of locations on which bit values of *s* are located. Then, by treating a given partial template *s* as a seed, we can now model the probabilistic space of iris templates that have the same partial template information as *s* with the Seeded Ising Model. Thus, we may reconstruct intruder templates from the partial template *s* by sampling iris templates from the space T(s) via the Metropolis algorithm [29] adapted for the Seeded Ising Model as stated in [8].

### 4.1. Sampling via Metropolis Algorithm

First note that, under the Seeded Ising Model, the probability P(x) is proportional to

$$\begin{array}{ccc} & & \exp\left(J_{v}\sum_{i{∼}_{v}j}x_{i}x_{j}+J_{h}\sum_{i{∼}_{h}j}x_{i}x_{j}\right)\\ & & =\exp\left(J_{v}\left(q_{1}q_{2}-q_{2}-2d_{x}^{v}\right)+J_{h}\left(q_{1}q_{2}-2d_{x}^{h}\right)\right), \end{array}$$

where $d_{x}^{v}$ denotes the number of disagreeing vertical edges in template *x* and $d_{x}^{h}$ denotes the number of disagreeing horizontal edges. Thus, P(x) is also proportional to the un-normalized probability π(x), which is given by $\pi \left(x\right)=\exp\left(-2J_{v}d_{x}^{v}-2J_{h}d_{x}^{h}\right).$

Let a template x∈T(s) be represented by a vector

$$x=(x_{1},x_{2},\cdots ,x_{k-1},x_{k},x_{k+1},\cdots ,x_{q_{1}q_{2}}).$$

Then, the Metropolis algorithm modified for our context would have the following steps:

1. Start with an initial template x∈T(s).
2. Select randomly a *non-seed index*
  $$k\in \{1,2,\cdots ,q_{1}q_{2}\}\backslash I.$$
3. Propose a new template $x^{\prime }$ as
  $$x^{\prime }=\left(x_{1},x_{2},\cdots ,x_{k-1},-x_{k},x_{k+1},\cdots ,x_{q_{1}q_{2}}\right).$$
4. Define the proposal probability of $t\to t^{\prime },$ moving from a template *t* to a template $t^{\prime }$ by
  $$Q\left(t\to t^{\prime }\right)=\left\{\begin{array}{ccc} \frac{1}{q_{1}q_{2}-\left|I\right|}, & & \mathrm{if}\;t,t^{\prime }\;\mathrm{differ}\;\mathrm{at}\;\mathrm{exactly}\\ & & \mathrm{one}\;\mathrm{non}-\mathrm{seed}\;\mathrm{index}.\\ 0, & & \mathrm{otherwise}. \end{array}\right.$$
  Then, accept $x^{\prime }$ with probability $A(x\to x^{\prime }),$
  $$A\left(x\to x^{\prime }\right)=\min\left[1,\frac{\pi \left(x^{\prime }\right)Q\left(x^{\prime }\to x\right)}{\pi \left(x\right)Q\left(x\to x^{\prime }\right)}\right],$$
  where π(x) is the un-normalized probability of x. Since $x,x^{\prime }$ differ in exactly one non-seed index *k* by the construction of $x^{\prime }$ from x,$Q(x\to x^{\prime })=$ $Q(x^{\prime }\to x)>0,$ we thus get
  $$A\left(x\to x^{\prime }\right)=\min\left[1,\frac{\pi (x^{\prime })}{\pi (x)}\right].$$
  The ratio in $A(x\to x^{\prime })$ is
  $$\begin{array}{ccc} & & \exp(-2J_{v}\left(d_{x^{\prime }}^{v}-d_{x}^{v}\right)-2J_{h}\left(d_{x^{\prime }}^{h}-d_{x}^{h}\right))\\ & & =\exp(2J_{v}\left(d_{x,k}^{v}-a_{x,k}^{v}\right)+2J_{h}\left(d_{x,k}^{h}-a_{x,k}^{h}\right)), \end{array}$$
  where $d_{x,k}^{v}$ is the number of disagreeing vertical edges between the index *k* and its vertically adjacent indices in template x, and $a_{x,k}^{v}$ is the number of agreeing vertical edges for *x* at k.$d_{x,k}^{h}$ and $a_{x,k}^{h}$ are defined similarly.
5. Generate a uniform random number u∈(0,1) and accept $x^{\prime }$ as the current template if $u<A(x\to x^{\prime }).$ Otherwise, keep *x* as the current template and go to Step 2.

### 4.2. Generating Intruder Templates

The procedure for obtaining an intruder template in [8] is briefly summarized here: Let $t^{0}$ be an initial template for the Metropolis algorithm, and $t^{n}$ be the template obtained after *n* iterations by the Metropolis algorithm. Then, for predetermined positive integers *L* and $n_{j}$’s for j=1,2,⋯,L with the condition that $0\leq n_{1}<n_{2}<\cdots <n_{L},$ we define the intruder template $r^{n}$ whose value $r_{i}^{n}$ at position *i* is given by the following:

(2) $$r_{i}^{n}=\left\{\begin{array}{cc} 1, & \;\mathrm{if}\;\sum_{j=1}^{L}t_{i}^{n_{j}}\geq 0\\ -1, & \;\mathrm{if}\;\sum_{j=1}^{L}t_{i}^{n_{j}}<0, \end{array}\right.$$

where n denotes the vector of $n=(n_{1},\cdots ,n_{L}).$ Note that in the above procedure, we need a partial template *s* initially, and the resulting templates of $t^{n}$ or $r^{n}$ naturally depend on s. To denote this dependency more clearly, we may use the notations of $t^{n}\left(s\right)$ or $r^{n}\left(s\right)$ in the subsequent discussion.

For real human iris templates, it is observed that the positions of bits whose values are 0 are randomly distributed over a template, and thereby the number of 0s in a template is about 50% of the total number of bits in the template. For this reason, the number of 0s in a partial template *s* is also about 50% of the total number of bits in *s* when *s* is obtained by the division methods of block-form or R-dispersion. This property still remains true for intruder templates if the partial template *s* has this property. However, intruder templates do not have such a property if almost all bit values of the partial template *s* are 0s (or 1s). Thus, we need additional treatment to generate reasonably realistic intruder templates when a partial template is obtained by the method of Z-Dispersion.

Let s:I→{−1,1} be a partial template obtained by the method of Z-Dispersion. To generate reasonably good intruder templates, first we randomly select a subset $I^{\prime }\subseteq \left\{1,2,\cdots ,mn\right\}$ so that $I\cap I^{\prime }=\emptyset$ and $\left|I|=\right|I^{\prime }|,$ where |A| denotes the cardinality of a set A. With this $I^{\prime },$*s* is extended to a partial template $s^{\prime }:I\cup I^{\prime }\to \left\{-1,1\right\}$ so that the restriction of $s^{\prime }$ on *I* is identical with *s*, i.e., $s=s^{\prime }{|}_{I}$ and the number of bits whose values are 0s is 50% of $|I\cup I^{\prime }|.$ This new partial template $s^{\prime }$ is called a *complementary* partial template and is denoted by $s^{\prime }=c\left(s\right).$ Then, intruder templates are generated from this complementary partial template c(s) via the same procedure as the above. By this process, we can obtain intruder templates $t^{n}\left(c\left(s\right)\right)$ or $r^{n}\left(c\left(s\right)\right).$ To simplify these notations, we denote intruder templates by $t^{n}\left(s\right)$ or $r^{n}\left(s\right)$ rather than $t^{n}\left(c\left(s\right)\right)$ or $r^{n}\left(c\left(s\right)\right)$ in the case where *s* is a partial template obtained by Z-Dispersion.

In the above procedure, we randomly select a complementary partial template c(s) to generate intruder templates. Thus, intruder templates $t^{n}\left(c\left(s\right)\right)$ or $r^{n}\left(c\left(s\right)\right)$ clearly depend on c(s), which may contain wrong information about the real template from which *s* came from. To minimize this effect, we may apply again the idea of bagging (**b**ootstrap **agg**regat**ing**) [30] in machine learning. In other words, we randomly select multiple complementary partial templates $c_{1}\left(s\right),c_{2}\left(s\right),\cdots ,c_{k}\left(s\right)$ rather than just one complementary partial template. Then, we define a intruder template $t^{n,k}\left(s\right)$ whose value at position *i* is given by

(3) $$t_{i}^{n,k}\left(s\right)=\left\{\begin{array}{cc} 1, & \;\mathrm{if}\;\sum_{j=1}^{k}t_{i}^{n}\left(c_{j}\left(s\right)\right)\geq 0\\ -1, & \;\mathrm{if}\;\sum_{j=1}^{k}t_{i}^{n}\left(c_{j}\left(s\right)\right)<0, \end{array}\right.$$

where *k* is the number of multiple complementary partial templates. Similarly, $r_{i}^{n,k}\left(s\right),$ the value of the intruder template $r^{n,k}\left(s\right)$ at position *i*, is also defined by

(4) $$r_{i}^{n,k}\left(s\right)=\left\{\begin{array}{cc} 1, & \;\mathrm{if}\;\sum_{j=1}^{k}r_{i}^{n}\left(c_{j}\left(s\right)\right)\geq 0\\ -1, & \;\mathrm{if}\;\sum_{j=1}^{k}r_{i}^{n}\left(c_{j}\left(s\right)\right)<0, \end{array}\right.$$

where n denotes the vector of $n=(n_{1},\cdots ,n_{L}).$

## 5. Intruder Attack Analysis

To analyze the integrity of the distributed template storage and comparison schemes depending on the division schemes and/or the size of partial templates, we performed statistical experiments. In the experiments, we used the algorithm developed by [31] for iris template generation with slight modification of template size. The size of iris templates generated by the modified algorithm is 8×256, and the size of the real (or imaginary) part of the templates is 8×128.

**Figure 6:**
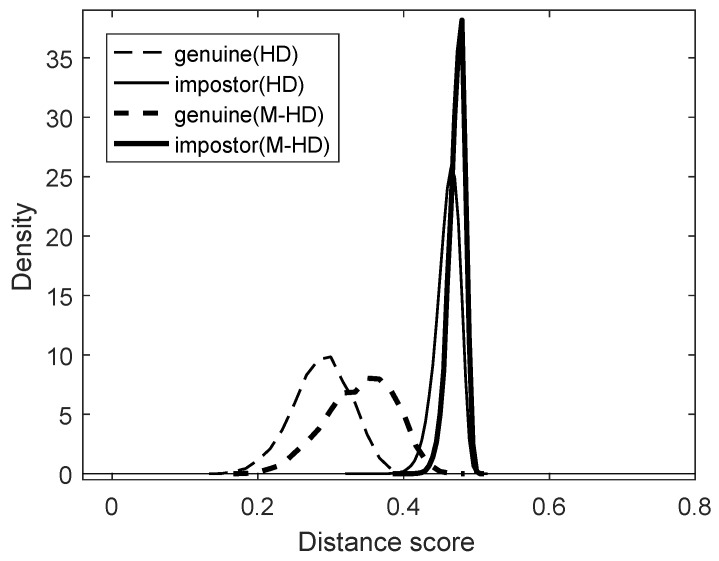
Distributions of genuine/impostor scores for the Hamming distance (HD) and the modified Hamming distance (M-HD).

The dataset used in this paper for statistical experiments consists of reasonably good images selected from ICE2005 Dataset, which was used for Iris Challenge Evaluation 2005 [32]. Since ICE2005 Dataset contains highly occluded images, we only selected images with a good enough iris pattern to minimize any side effects. Table 1 shows basic statistics of the ICE2005 Dataset, and Table 2 shows basic statistics of the dataset used in this paper. Figure 6 shows the comparison results of the dataset for the two dissimilarity metrics: the Hamming distance and the modified Hamming distance.

From the distributions in Figure 6, we may observe that the most genuine comparison has a Hamming distance less than 0.4, and most impostor comparison has a Hamming distance greater than 0.4. Therefore, roughly speaking, the best threshold that distinguishes whether a given pair of iris templates comes from the same person or not would be around 0.4 when Hamming distance is used as a metric. However, when the modified Hamming distance is used, the best threshold would be a little bigger since the modified Hamming distance is higher than the Hamming distance when the Hamming distance is less than 0.5. Table 3 shows the False Match Rate and False Non-Match Rate when Hamming distance (HD) and modified Hamming distance (M-HD) are used as a metric for each value of thresholds 0.4 and 0.431. From the table, we may choose the threshold value of 0.431 for the modified Hamming distance as a metric as a comparable threshold for the threshold value of 0.4 for the Hamming distance as a metric. For reference, Table 3 also shows the Equal Error Rate (EER) for HD and M-HD.

**Figure 7:**
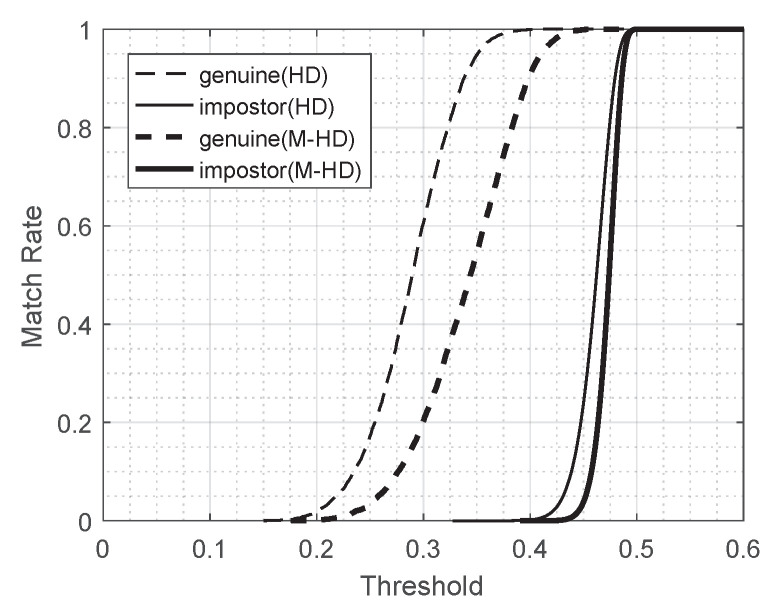
Match Rates.

With a match rate m(d) defined as the ratio of the number of comparison trials with Hamming distance (or modified Hamming distance) less than or equal to d,

$$m\left(d\right)=\frac{\left|\{i|d_{i}\leq d\}\right|}{M}$$

for a given collection of *M* comparison scores $(d_{1},d_{2},$$\cdots ,d_{M}),$ the differences in the characteristics of the Hamming distance and the modified Hamming distance are shown as Figure 7. In fact, the match rate m(d) is the empirical cumulative distribution of a collection of comparison scores. Figure 7 shows match rate curves for several different collections of comparison scores. The thin and thick dashed curves represent the match rates for genuine comparisons of 948 images with the Hamming distance and the modified Hamming distance used, respectively. The thin and thick solid curves represent the match rates for impostor comparisons of the same dataset with the Hamming distance and the modified Hamming distance used, respectively. (See Table 2 for the details of the number of comparisons.) Note that dashed curves represent the positive match rates, and solid curves show the false match rates.

For the statistical experiments, 948 templates from the 948 images are divided into partial templates by each scheme of three division methods of block-form division (B), R-dispersion (R-D), and Z-dispersion (Z-D), with three different sizes of partial templates: 1/4, 1/8, and 1/16 of the undivided full template size. For each case of division method and the size of partial templates, one partial template is selected for each full template, and 10 intruder templates $t^{n}$ (or $t^{n,k}$) and 10 intruder templates $r^{n}$ (or $r^{n,k}$) are generated. For the case of Z-dispersion division, 100 intruder templates of $t^{n,1}$ and 100 of $r^{n,1}$ are also additionally generated for each partial template. In the generation of intruder templates, parameters of n,n,k used are as follows: $n=10^{5},$$n=10^{3}\left(1,2,\cdots ,10^{2}\right),$ and k=10.

In the experiments, we suppose two different scenarios: one is the case where a partial template *s* of a full template *x* is leaked, and *x* is not replaced in the iris recognition system; thus intruder templates reconstructed from *s* are compared with x. The other scenario is the case where a partial template *s* of a full template *x* is leaked, and *x* is replaced by another template $x^{\prime }$ of the same person. Thus, intruder templates reconstructed from *s* are compared with $x^{\prime }.$ The first scenario is simply denoted by *Leaked Template Not Replaced*, and the second scenario by *Leaked Template Replaced*. In order to perform statistical experiments for the scenario of Leaked Template Not Replaced, each intruder template from a partial template *s* is compared with the original template *x* from which *s* is taken, and Hamming distance (HD) and the modified Hamming distance (M-HD) are computed for the dissimilarity scores. This distance score is called intruder distance score. For the other scenario of Leaked Template Replaced, each intruder template from *s* is compared with a template $x^{\prime }$, which is another template of the same person to whom *x* belongs. Table 4 shows the numbers of intruder comparisons for each case. To compare the safety of distributed template storage and comparison schemes when partial templates are leaked, we define the Intruder Match Rate, IMR(*d*) as the match rate m(d) of the collection of intruder distance scores for a threshold d.

Table 5 shows intruder match rates in all cases with threshold 0.4. For each case, IMR(*d*) is computed for the Hamming distances (HD) of intruder comparison. In addition, IMR(*d*) for the modified Hamming distances (M-HD) of intruder comparison is computed. Table 6 also shows intruder match rates with threshold 0.431.

From the intruders’ point of view, they will try to intrude into an iris recognition system using reconstructed iris templates with the highest IMR when a partial template *s* is available. Thus, for the division methods of block-form and R-dispersion, intruders will try to intrude into the system with $r^{n},$ and with $r^{n,k}$ for the division method of Z-dispersion. Thus, the safety of distributed template storage and comparison schemes may be measured by the IMR of $r^{n}$ or $r^{n,k}$: the lower the IMR is, the safer the system is. For a given scheme of dividing a full template into partial templates including the method of division and the size of partial templates, the IMR of $r^{n}$ or $r^{n,k}$ is defined as the *risk of leakage* for the scheme. Except for the scheme *B16* of the block-form, division in which the full template is divided into 16 partial templates, the risk of leakage is more than 3%, which is not acceptable for systems of high security, when the system uses Hamming distance as a metric with the threshold of 0.4 and a leaked template is not replaced in the system. For the scheme of B16, the same risk of leakage is 0.0527%, which is less than 0.1766% of False Match Rate in Table 3. This implies that the system of distributed template storage and comparison with the scheme of B16 is safer than most iris recognition systems, even when a partial template is leaked and it still remains in the system in terms of Hamming distance with the threshold of 0.4.

However, the system with Hamming distance as a metric is vulnerable to the occlusion attack as stated in Section 3. For this reason, we suggest the modified Hamming distance be used in the system of high security. When using the modified Hamming distance in the system, the threshold should be adapted properly to maintain the system’s operating characteristics, for example, the False Match Rate. From Table 3, the threshold of 0.431 for the modified Hamming distance would be equivalent to the threshold of 0.4 for Hamming distance for the dataset we used in terms of FMR. Table 6 shows that with this threshold value and the modified Hamming distance, the scheme of B16 is still found to be as secure as usual iris recognition systems with the same metric and the same threshold even when a leaked partial template is not replaced.

**Figure 8:**
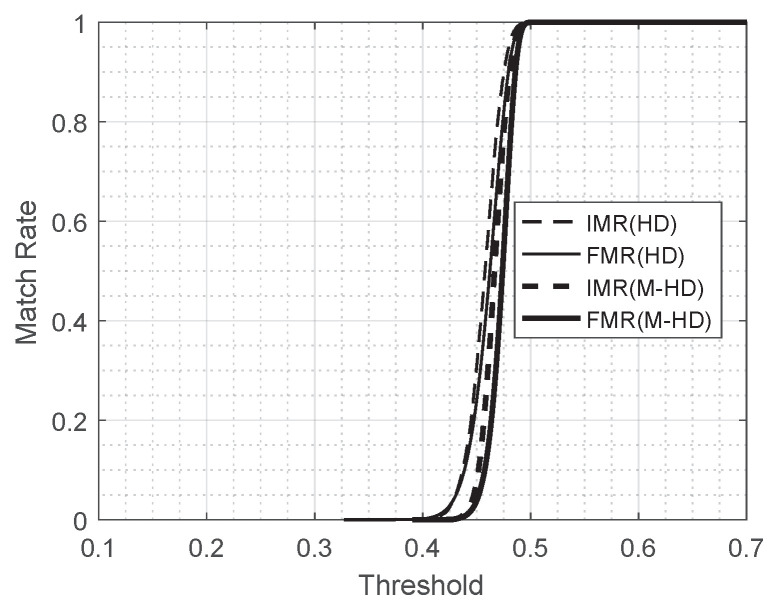
IMR of $r^{n}$ (B16, Leaked Template Not Replaced) and FMR.

**Figure 9:**
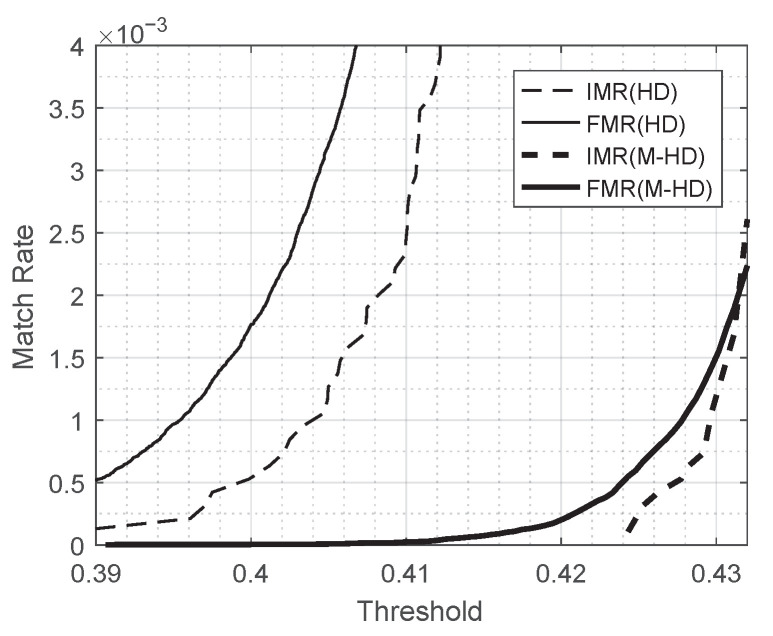
IMR of $r^{n}$ (B16, Leaked Template Not Replaced) and FMR (Zoomed).

This observation remains true for different values of threshold when it is in a reasonably good range. Figure 8 shows the risk of leakage for the scheme B16 for different values of threshold compared with FMR at the corresponding threshold. In Figure 8, the thin and thick solid curves represent FMR measured by Hamming distance and FMR by the modified Hamming distance, respectively, and the thin and thick dashed curves represent the risk of leakage for the scheme B16 with Hamming distance and with the modified Hamming distance used, respectively, in the scenario of Leaked Template Not Replaced. FMR and the risk of leakage looks comparable for all values of threshold for both of metrics, Hamming distance and the modified Hamming distance. Figure 9 is the magnified figure of Figure 8 around the range of threshold [0.39,0.432]. It shows that the risk of leakage for the scheme of B16 is less than or comparable with FMR for all values in the range of threshold.

## 6. Conclusions and Policy Recommendations

In this paper, we study the safety issues concerning the schemes of distributed iris template storage and comparison.

In particular, we investigate the risk of leakage for several schemes: all the combinations of division methods including block-form division, R-dispersion, Z-dispersion. and the sizes of subtemplates including 1/4, 1/8, and 1/16. To analyze the safety issues that arise when a partial template is leaked, multiple methods of generating intruder templates from a leaked partial template are proposed based on the Seeded Ising Model, and Intruder Match Rates are measured for every case of schemes in two different scenarios: Leaked Template Not Replaced and Leaked Template Replaced. Statistical experiments show that the scheme of B16 is as secure as most iris recognition systems with no leaked templates even when partial templates leaked in one location are not replaced.

We also propose the modified Hamming distance against the occlusion attack. From the analysis of our experiments, the system of distributed template storage and comparison is recommended to use the scheme of B16 with the modified Hamming distance as a metric.

There have been many studies including the approaches of cancelable biometric and the methods of biometric cryptosystem to resolve the security issues of biometric systems. Our methods and the current state-of-the-art schemes are not in any way contradictory or in competition, but rather complementary. In other words, the methods of cancelable biometrics or the biometric cryptosystems can be applied to our distributed template storage and comparison to further enhance its security.

## Figures and Tables

**Table 1:** ICE2005 Dataset used (with error corrected).

Position	# of Images	# of Subjects
Left	1527	119
Right	1426	124
Total	2953	132

**Table 2:** Dataset used in this paper.

Position	# of Images	# of Subjects
Right	948	120
	# of Genuine	# of Impostor
	comparisons	comparisons
	5953	442,925

**Table 3:** False Match Rate (FMR), False Non-Match Rate (FNMR), and Equal Error Rate (EER) for the chosen subset of 948 images from the ICE2005 Dataset.

	Threshold	HD	M-HD
FMR	0.4	0.1766%	0.0002%
FNMR	0.4	0.0672%	9.2726%
FMR	0.431	5.0555%	0.1736%
FNMR	0.431	0.0000%	0.9911%
EER		0.1176%	0.5112%

**Table 4:** The numbers of intruder comparisons for Leaked Template Not Replaced (A) and Leaked Template Replaced (B).

	Intruder	A	B
B	$t^{n}$	9480	119,060
	$r^{n}$	9480	119,060
R-D	$t^{n}$	9480	119,060
	$r^{n}$	9480	119,060
Z-D	$t^{n,1}$	94,800	1,190,600
	$r^{n,1}$	94,800	1,190,600
	$t^{n,k}$	9480	119,060
	$r^{n,k}$	9480	119,060

**Table 5:** Intruder Match Rate with threshold 0.4.

	Intruder	1/4		1/8		1/16	
		HD	M-HD	HD	M-HD	HD	M-HD
Leaked Template Not Replaced							
B	$t^{n}$	58.88%	37.42%	3.587%	0.0105%	0.0211%	0.0000%
	$r^{n}$	60.63%	38.52%	4.430%	0.0738%	0.0527%	0.0000%
R-D	$t^{n}$	100.0%	84.96%	12.04%	1.551%	0.0105%	0.0000%
	$r^{n}$	100.0%	99.30%	93.44%	48.05%	3.112%	0.3165%
Z-D	$t^{n,1}$	100.0%	96.58%	24.80%	4.710%	0.0116%	0.0000%
	$r^{n,1}$	100.0%	99.80%	95.52%	51.54%	3.4251%	0.3787%
	$t^{n,k}$	100.0%	99.89%	98.92%	60.06%	0.9916%	0.0527%
	$r^{n,k}$	100.0%	100.0%	100.0%	85.89%	45.70%	14.01%
Leaked Template Replaced							
B	$t^{n}$	0.2604%	0.0529%	0.0050%	0.0000%	0.0000%	0.0000%
	$r^{n}$	0.3444%	0.0655%	0.0067%	0.0000%	0.0008%	0.0000%
R-D	$t^{n}$	13.8896%	2.4223%	0.0336%	0.0000%	0.0000%	0.0000%
	$r^{n}$	78.8443%	33.9929%	10.8819%	1.7907%	0.1050%	0.0017%
Z-D	$t^{n,1}$	33.1925%	8.7592%	0.0463%	0.0008%	0.0004%	0.0000%
	$r^{n,1}$	77.5003%	33.4300%	7.5040%	1.1236%	0.0832%	0.0011%
	$t^{n,k}$	73.6377%	31.1272%	3.9325%	0.5669%	0.0067%	0.0000%
	$r^{n,k}$	89.9177%	47.7961%	38.7410%	10.6392%	1.8806%	0.1755%

**Table 6:** Intruder Match Rate with threshold 0.431.

	Intruder	1/4		1/8		1/16	
		HD	M-HD	HD	M-HD	HD	M-HD
Leaked Template Not Replaced							
B	$t^{n}$	84.00%	66.45%	53.06%	18.71%	3.723%	0.0527%
	$r^{n}$	84.46%	66.94%	57.55%	22.90%	5.253%	0.1477%
R-D	$t^{n}$	100.00%	99.60%	85.47%	43.31%	3.914%	0.5380%
	$r^{n}$	100.00%	100.00%	99.99%	92.25%	49.61%	18.37%
Z-D	$t^{n,1}$	100.00%	99.99%	93.55%	55.96%	4.561%	0.5717%
	$r^{n,1}$	100.00%	100.00%	99.98%	92.63%	51.40%	19.19%
	$t^{n,k}$	100.00%	100.00%	100.00%	94.66%	48.69%	16.96%
	$r^{n,k}$	100.00%	100.00%	100.00%	99.65%	96.85%	65.87%
Leaked Template Replaced							
B	$t^{n}$	11.86%	4.581%	1.470%	0.0949%	0.1688%	0.0008%
	$r^{n}$	13.02%	5.295%	2.128%	0.1697%	0.3309%	0.0017%
R-D	$t^{n}$	71.50%	29.89%	6.197%	0.6938%	0.2318%	0.0050%
	$r^{n}$	98.26%	76.62%	63.52%	25.12%	7.778%	1.175%
Z-D	$t^{n,1}$	86.31%	46.65%	7.977%	1.178%	0.2572%	0.0035%
	$r^{n,1}$	97.99%	74.68%	56.50%	20.68%	6.949%	0.9154%
	$t^{n,k}$	97.63%	71.76%	50.02%	16.96%	2.307%	0.2217%
	$r^{n,k}$	99.35%	83.97%	88.49%	50.04%	34.73%	9.965%

## Data Availability

ICE2005 Dataset is provided by NIST for the Iris Challenge Evaluation (ICE) 2005. Refer to https://www.nist.gov/publications/iris-challenge-evaluation-2005 for more details. For information on obtaining the ICE 2005 Dataset and challenge problem see http://iris.nist.gov/ice.
